# Activation of Pax7-Positive Cells in a Non-Contractile Tissue Contributes to Regeneration of Myogenic Tissues in the Electric Fish *S. macrurus*

**DOI:** 10.1371/journal.pone.0036819

**Published:** 2012-05-31

**Authors:** Christopher M. Weber, Mark Q. Martindale, Stephen J. Tapscott, Graciela A. Unguez

**Affiliations:** 1 Biology Department, New Mexico State University, Las Cruces, New Mexico, United States of America; 2 Kewalo Marine Lab, University of Hawaii, Honolulu, Hawaii, United States of America; 3 Division of Human Biology, Fred Hutchinson Cancer Research Center, Seattle, Washington, United States of America; Brigham & Women’s Hospital - Harvard Medical School, United States of America

## Abstract

The ability to regenerate tissues is shared across many metazoan taxa, yet the type and extent to which multiple cellular mechanisms come into play can differ across species. For example, urodele amphibians can completely regenerate all lost tissues, including skeletal muscles after limb amputation. This remarkable ability of urodeles to restore entire limbs has been largely linked to a dedifferentiation-dependent mechanism of regeneration. However, whether cell dedifferentiation is the fundamental factor that triggers a robust regeneration capacity, and whether the loss or inhibition of this process explains the limited regeneration potential in other vertebrates is not known. Here, we studied the cellular mechanisms underlying the repetitive regeneration of myogenic tissues in the electric fish *S. macrurus*. Our *in vivo* microinjection studies of high molecular weight cell lineage tracers into single identified adult myogenic cells (muscle or noncontractile muscle-derived electrocytes) revealed no fragmentation or cellularization proximal to the amputation plane. In contrast, ultrastructural and immunolabeling studies verified the presence of myogenic stem cells that express the satellite cell marker Pax7 in mature muscle fibers and electrocytes of *S. macrurus*. These data provide the first example of Pax-7 positive muscle stem cells localized within a non-contractile electrogenic tissue. Moreover, upon amputation, Pax-7 positive cells underwent a robust replication and were detected exclusively in regions that give rise to myogenic cells and dorsal spinal cord components revealing a regeneration process in *S. macrurus* that is dependent on the activation of myogenic stem cells for the renewal of both skeletal muscle and the muscle-derived electric organ. These data are consistent with the emergent concept in vertebrate regeneration that different tissues provide a distinct progenitor cell population to the regeneration blastema, and these progenitor cells subsequently restore the original tissue.

## Introduction

A major challenge in the study of mammalian regeneration remains the identification of the limiting factors that constrain the restoration of a tissue with the purpose of modifying these factors to boost the regeneration potential [1], [2]. Many vertebrate animals repair and remodel their injured or lost tissues primarily through the activation of stem cells and/or the process of dedifferentiation, i.e., a process by which mature cells lose their differentiated phenotype to produce progenitor cells that can reenter the cell cycle. Multiple regeneration mechanisms may come into play in one animal in response to injury or disease. For example, in humans liver tissue regenerates through a dedifferentiation process [3], whereas skeletal muscle regenerates through the activation of myogenic stem cells [4], [5] suggesting that programs of tissue regeneration might be broadly conserved but their expression is regulated by tissue-specific constraints.

Although one mechanism might be favored under certain conditions, it is not possible to exclude some contribution from an alternative mode of regeneration. Nevertheless, the idea that a greater potential for regeneration is associated with a corresponding potential for cell dedifferentiation remains broadly accepted [6], [7], [8]. Support for this idea is based largely on the vast body of evidence from experiments in urodele amphibians such as the newt and axolotls, which are regarded as the champions of regeneration among vertebrates. Urodeles are able to regenerate limbs, tail, jaws, ocular tissues like retina and lens, and some portions of the heart. Studies have suggested that their ability to regenerate these tissues depends largely on dedifferentiation of cells at the site of injury to reenter the cell cycle [6], [8]. In contrast, there is less evidence for the existence of adult stem cell populations and their contribution to restored tissues in urodeles [9], [10].

Some studies using urodele amphibians have indicated that regeneration does not proceed in the absence of cell dedifferentiation [6], [11], [12], [13]. Whether cell dedifferentiation is the fundamental factor that triggers a robust regeneration capacity, and whether the loss or inhibition of this process explains the limited regeneration potential in other vertebrates is not known. In order to test this idea by studying different species, it is important to first recognize that cell dedifferentiation involves different (and likely, independent) cellular events [14]. The first involves the reentry of myonuclei into the cell cycle suggesting that the postmitotic arrest of nuclei in mature muscle fibers is reversible in urodeles [15]. The second type of dedifferentiation involves the reversal of multinucleated muscle fibers to mononucleated cells (aka, cellularization). The third event involves the loss of gene expression markers of the differentiated state such as sarcomeric proteins. Each of these processes has been demonstrated to occur in the dedifferentiation of skeletal muscle fibers after limb amputation in urodeles. However, the extent to which each of these events occurs in response to injury among other vertebrates has not been well studied. Investigation of the regenerative processes in vertebrates other than urodeles with extensive regenerative capacities is essential for the identification of the limiting factors that constrain the restoration of tissues in some species and development of strategies to improve their regeneration potential.

The South American gymnotiform electric fishes are unique vertebrate teleosts in that they possess a muscle-derived electric organ (EO) that is specialized for the production of an electric field outside the body [16] and they also possess a robust capacity to regenerate lost tissues. Of the seventy known species of gymnotiforms, all can regenerate their tails following amputation. Specifically, they can replace lost spinal cord, skin, electroreceptors, skeleton, blood vessels, skeletal muscle and the muscle-derived electric organ [17], [18], [19], [20], [21], [22], [23], [24]. Tail regeneration in all gymnotiforms begins by epidermal cells covering the wound followed by formation of a blastema of apparently undifferentiated cells appearing as a small swelling at the end of the tail that gives rise to all newly formed adult tissues [25], [18], [19], a process widespread in the animal kingdom, appearing in both invertebrate and vertebrate organisms [26], [27]. Moreover, some gymnotiforms can replace all tissues lost after repeated tail amputations, suggesting an inexhaustible regeneration capacity in the adult [28] and representing the ideal experimental system for investigation of regeneration in teleosts.

**Figure 1:**
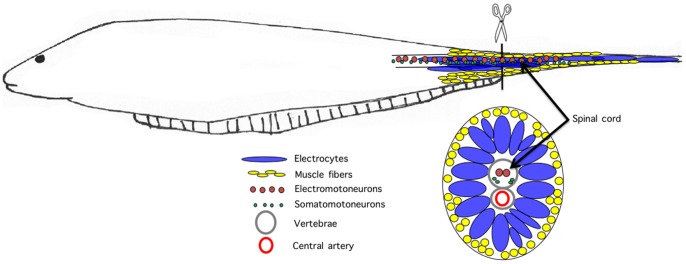
Schematic illustration of *S. macrurus* and the amputation protocol used in the present study. Tails were amputated at the end of the ventral fin (scissors). At the site of amputation, vertebrae, central artery, motor neurons, electrocytes and muscle fibers are present. The spinal cord contains two distinct populations of motor neurons: electromotoneurons that innervate electrocytes and somatomotoneurons that innervate muscle fibers. Muscle fibers are located peripherally and surround a central core of electrocytes. Electrocytes are up to 3 mm in length with a cross sectional area up to 30 times that of the adjacent muscle fibers. As shown in the cross sectional view at the bottom, muscle fibers and electrocytes are separated into distinct compartments. In this study, we analyzed the most-distal segment (50 mm) of the tail stump after tail amputation to investigate effects of amputation on tissues immediately adjacent to the injury site.

Tail regeneration in gymnotiforms has been most studied in the yellow stripe knife fish *S. macrurus* [21], [28], [29]. As in all gymnotiforms, *S. macrurus* responds to tail amputation by wound healing, blastema formation, and blastema elongation by cell proliferation. At the site of amputation at the end of the ventral fin, electrocytes predominate, but also present are skeletal muscle fibers found peripherally beneath the skin and vertebrae protecting the central artery and motoneurons that innervate electrocytes and muscle fibers (Figure 1). Cell differentiation during regeneration proceeds with slow-twitch fibers appearing under the skin followed by differentiation of fast-twitch fibers medial to the slow fibers [21]. A subpopulation of the most centrally-located fast-twitch fibers then fuses and subsequently downregulates many muscle genes to give rise to cells of the electric organ, i.e., electrocytes, all while the spinal cord, vertebrae and blood vessel are differentiating within the center of the tail [21], [28]. Within three weeks of tail amputation, a new tail develops complete with peripheral slow and fast muscle fibers and a centrally located electric organ surrounding the vertebrae, spinal cord and central blood vessel [28].

A previous study looking at the labeling patterns of cell replication markers during tail regeneration in *S. macrurus* suggested that the source of the regeneration blastema consisted of local reserve cells that became activated to contribute to the restoration of all lost tissues [21]. Specifically, the authors concluded that myogenic satellite cells contributed to the blastema and the regeneration of skeletal muscle and the myogenically derived EO reviving the argument for determining the extent to which stem cell activation and dedifferentiation are conserved across different vertebrate species. Here we tested the hypothesis that myogenic stem cells contribute to blastema formation during adult tail regeneration in *S. macrurus*. We detected myogenic cells associated with adult skeletal muscle and electric organ that express the mammalian satellite cell marker Pax7 [30]. After tail amputation, these Pax7-positive satellite cells proliferated and populated blastema regions that exclusively regenerate muscle fibers and electrocytes. Accompanying *in vivo* microinjection studies using high molecular weight lineage tracers showed that multinucleate muscle fibers and electrocytes proximal to the injury site do not undergo fragmentation (cellularization) and do not participate in the formation of regenerated myogenic cells. Our results reveal a regeneration process in *S. macrurus* that is dependent on the activation of myogenic stem cells for the renewal of both skeletal muscle and the muscle-derived electric organ. These data also suggest that muscle dedifferentiation is not required for a robust regenerative capacity in the myogenic lineage of the adult teleost *S. macrurus*.

## Materials and Methods

### Animal Surgeries and Tissue Preparation

*S. macrurus* is a fresh-water species of knife fish native to South America and was obtained commercially from Segrest Farms (Gibsonton, FL). Adult fish, 20–35 cm in length, were housed individually in 15 to 20-gallon aerated aquaria maintained at 25–28°C and fed three times weekly. Fish were anesthetized in 2-phenoxyethanol (1∶1,500 in tank water) and a tail segment distal to the ventral fin was amputated with a sharp blade as described previously [28], [29], [31]. In one group the most-distal segment (50 mm) of the tail stump along with the blastema was harvested 7 (n = 4) and 14 (n = 3) days after initial tail amputation. In a second group, only the regeneration blastema was removed 1 (n = 5) or 2 (n = 5) weeks after initial tail amputation. In a third group, only the distal-most section of ventral muscle (2–3 cm) was amputated. In this procedure, electric organ, central blood vessel and spinal cord were left intact and regeneration studied until full restoration of muscle was complete. Immediately following each amputation surgery, fish were treated with a topical antibiotic (Nystatin and Triamcinolone Acetonide Ointment USP), returned to their tanks, Stress Coat® (Aquarium Pharmaceuticals, Inc., Chalfont, PA) was added to the aquarium tank as an additional anti-infection agent, and fish were monitored until they recovered fully from anesthesia. Control fish were overdosed in 2-phenoxyethanol (1∶500 in tank water) and the liver, brain, spinal cord, ventral skeletal muscle and EO were excised under a dissecting microscope. All procedures used in this study followed the American Physiological Society Animal Care Guidelines and were approved by the Animal Use Committee at New Mexico State University. Excised tail segments were 1) immediately immersed in RNA*later™* (Ambion, Austin, TX) and stored at −80°C until RNA extraction, 2) frozen directly in liquid nitrogen and stored at −80°C until protein extraction for Western blots, or 3) frozen on cork with Tissue Freezing Medium™ (TBS™, Durham, NC) in isopentane chilled in liquid nitrogen and used for immunohistochemical analysis.

### RNA Isolation and Reverse-transcription and Polymerase Chain Reaction (RT-PCR)

Total cellular RNA was isolated from skeletal muscle, EO, spinal cord, brain, and liver of adult control fish using a protocol adapted from the guanidinium isothiocyanate method used in previous studies with *S. macrurus* myogenic tissues [31]–[33]. To isolate total RNA from distal-most (50 mm) tail stumps and regeneration blastema (1- and 2-week), we used the RNeasy kit (Qiagen, Valencia, CA). To remove residual DNA, total RNA isolated from each tissue sample was treated with DNase I, Amplification Grade (Ambion**,** Austin, TX) and analyzed by spectrophotometry (OD_260_/OD_280_). On average, total RNA isolations yielded 1–3% of starting material from each of the different tissues. All RNA samples were stored at −80°C.

### Cloning and Isolation of S. macrurus Pax7

Heterologous degenerate oligonucleotide primers were designed for Pax7 based on respective GenBank vertebrate sequences using Dialign and CODEHOP. Pax7 primers corresponding to the PAPGQNYPRT (sense primer) and GKKDDDDDC (antisense primer) domains were designed using alignments from protein sequences of *Danio rerio* (GenBank: NP_571400), *Salmo salar* (GenBank: CAF02090.1), *Tetraodon nigroviridis* (GenBank: CAG07040.1), and two sequences from *Salvelinus alpinus* (GenBank: CAG25716.1; CAG25714.1). To clone partial Pax7 cDNAs from *S. macrurus*, we carried out reverse transcription (RT) and PCR separately. The RT reaction from 250–300 ng total RNA was performed for 50 min at 42°C using the SuperScript™ First-Strand Synthesis System for RT-PCR (Invitrogen, Carlsbad, CA). PCR amplification (1 µg of 1-week regeneration blastema cDNA) was carried out using PCR with annealing temperature 5°C below the calculated T_m_ for 35–40 cycles with Platinum® *Taq* DNA Polymerase (Invitrogen).

RT-PCR products for Pax7 were PCR purified by gel extraction (Qiagen, Valencia, CA), subcloned into the Topo TA2.1 Plasmid (Invitrogen) or pGEMTeasy (Promega, Madison, WI) and transformed into Mach1 cells (Invitrogen) or JM109 cells (Promega). Plasmids from 10–20 cDNA clones of each transcript were isolated using the QIAprep® Spin Miniprep Kit (Qiagen), sequenced in both directions using a Li-Cor 4200 L Global IR2 DNA Sequencer or an Applied Biosystems automated DNA sequencer (Model 3100), and analyzed using the Vector NTI Suite 8.0 software (InforMax, Inc., Bethesda, MD). Upon verification of the cloned sequences obtained from 1-wk cDNA regeneration blastema, nondegenerate primers for the Pax7 transcript specific to *S. macrurus* were generated from the on-line Primer3 software program (http://frodo.wi.mit.edu/cgi-bin/primer3/primer3_www.cgi).

### Molecular Cloning of Full-length Pax7 from S. macrurus

RACE first-strand synthesis from total RNA (1 µg) of 1-week regeneration blastema was performed using the BD SMART™ RACE cDNA Amplification Kit (BD Biosciences). Pax7-specific primers used for 5′-RACE were 5′-GCACACTCCGTCCTTCAGCAGCTTG-3′ and 5′ - GCTGGAATGGCTACTTTACCG 3′, and for 3′-RACE were 5′-GAGACGGGTTCGATTCGTCCTGGAG–3′ and 5′– TAGCCAGGTAGTCCACAGCAC –3′. Purified products from Pax7 RACE experiments were subcloned into the pCR® 2.1-TOPO® vector (Invitrogen), transformed into One Shot® MACH1™ T1 Phage-Resistant Chemically Competent *E. coli* cells (Invitrogen), isolated with the QIAprep® Spin Miniprep Kit (Qiagen), and sequenced in both directions using either a Li-Cor-4200 L Global IR2 DNA or an Applied Biosystems automated DNA sequencer (Model 3100). Chromatogram traces were analyzed using the Vector NTI Suite 8.0 software (InforMax Inc.). Searches were performed using the Basic Local Alignment Search Tool (BLAST) network service from the National Center for Biotechnology Information (NCBI) (http://www.ncbi.nlm.nih.gov). Multiple sequence alignments were generated with ClustalW from the European Bioinformatics Institute (http://www.ebi.ac.uk/clustalw). The novel sequence described here and a transcript variant were submitted to NCBI and assigned accession numbers EU624121.2 and EU624121.1 for *S. macrurus* Pax7.

### Western Blotting

Fresh-frozen tissue samples were pulverized with a mortar and pestle in liquid nitrogen and total protein lysates were prepared using lysis buffer (1% Igepal CA-630, 0.5% sodium deoxycholate, 0.1% SDS, 10 mM beta-mercaptoethanol, 10 µg/mL PMSF, 5 µg/mL aprotinin, 0.1 mg/mL benzamidine, 1 µg/mL pepstatin A, 1 µg/mL leupeptin, 100 mM sodium orthovanadate, 15 µL/mL Triton X-100®, in Phosphate Buffered Saline (PBS) pH 7.4) on ice for 45 min and homogenized by two 15-sec pulses. Lysates were centrifuged at 14,000 g for 10 min at 4°C and supernatants were collected. Protein concentration was determined using the Bradford assay (Bio-Rad, Hercules, CA).

Proteins (20 µg/lane) were separated by SDS-PAGE alongside Kaleidoscope pre-stained standards on 4–15% Tris-HCl gradient gels (Bio-Rad; 100 V, 120 min) and transferred onto PVDF membranes using a Transblot SD semi-dry electrophoretic transfer cell (Bio-Rad) following manufacturer’s instructions. Membranes were washed twice with TBS-T [0.1% Tween-20 in Tris buffered saline (TBS)], blocked in blocking buffer (3% non-fat dry milk in TBS-T) for 60 min at RT, and incubated with anti-Pax7 antibody (1∶50; Developmental Hybridoma Bank, Iowa City, IA) diluted in 5% BSA in TBS-T overnight at 4°C. After PBST washes, membranes were incubated with an HRP-conjugated secondary antibody for 1 hr. The BioRAD amplification module was used according to manufacturer’s instructions. Colorimetric detection via BioRAD’s Opti-4CN™ Substrate Kit was performed according to manufacturer’s instructions to visualize antigen-antibody complexes.

### In vitro Translation of S. macrururs Pax7 cDNA

To further verify antibody specificity to *S. macrurus* Pax7 protein, a 1,435 bp Pax7 fragment from muscle was ligated into the pGEMTeasy vector, (Promega), and subcloned. Following sequence verification, the insert from muscle cDNA was translated *in* *vitro* using the TNT® T7 Coupled Reticulocyte Lysate System (Promega) following manufacturer’s instructions. Translation product was then separated by SDS PAGE (product diluted 4× in 2× SDS load buffer & 20 µL, 25 µL, and 30 µL were loaded), transferred to PVDF, and probed with the Pax7 antibody as described above.

### Electron Microscopy

Normal adult tails (n = 2) were examined under a transmission microscope (TEM). After surgical removal, blastemas and tails were fixed in 4% paraformaldehyde/2.5% gluteraldehyde overnight, transferred to sodium phosphate buffer (pH = 7.2), post-fixed in osmium (2% osmium in sodium phosphate buffer) for 1 hour, dehydrated in an alcohol series and propylene oxide, and embedded in Spurrs’ plastic resin (Polysciences, Warrington, PA). Ultrathin (90-nm) tissue cross sections were cut with a diamond knife, stained with uranyl acetate and lead citrate, and examined with the Hitachi HU11-E TEM (Cell Research Institute of the University of Texas at Austin).

### Imunofluorescence

Serial transverse and longitudinal cryosections (14–30 µm thick) were mounted on glass slides (Superfrost Plus, Fisher, Pittsburgh, PA) and air-dried at room temperature. Sections were surrounded with a hydrophobic barrier by using an ImmEdge pen (Vector Laboratories, Ingold, CA) to retain incubating solutions on the tissue. Sections were post-fixed on slides by immersion in 4% buffered formaldehyde (pH 7.4, 20 min, 37°C) and rinsed twice in 0.1 M PBS (pH 7.4). Slides were immersed in permeabilization solution (0.5% Triton® X-100 in PBS for 30 min.) and subsequently incubated in 1% blocking reagent (TSA kit, Invitrogen) for 1 hr at room temperature. Mouse monoclonal antibodies against Pax7 (1∶20), sarcomeric myosin heavy chain isoforms (MF20, 1∶100), and laminin (1∶100) were diluted in 1% blocking solution and applied to separate slides for 15–20 h at room temperature. Antibodies against Pax7 and MF20 were obtained from the Developmental Hybridoma Bank (Iowa City, IA) and the anti-laminin antibody was obtained from Sigma (St. Louis, MO). Tissue sections were incubated in 1% blocking solution without primary antibodies to assess nonspecific labeling by secondary antibodies. Sections were rinsed in PBS and incubated in horseradish peroxidase-conjugated goat anti-mouse IgG (TSA kit, diluted 1∶100 in 1% blocking solution) for 1 h. Tissue sections were rinsed in PBS, and incubated in Alexa Fluor 488 or 546 tyramide working solution (TSA kit, 10 min., diluted 1∶1000 in 0.0015% H_2_O_2_). Sections were rinsed in PBS, counterstained with propidium iodide (Sigma; 1∶1000, 1 min) or SYTOX Green nucleic acid stain (Invitrogen, 1∶1000, 1 min), rinsed in deionized water, and cover-slipped with Vectashield Hard Set mounting media (Vector) or Gelmount (Biomeda, Foster City, CA).

### BrdU Immunolabeling

Fish were given intraperitoneal injections of BrdU (Sigma) at a dose of 100 mg/kg. Fish were given two BrdU injections: immediately after tail amputation and 24 h after tail amputation. In these fish, the distal-most 50 mm portion of the tail stump was removed 7 or 14 days post amputation. For each group, non-amputated control fish were injected (n = 2) to determine the level of cell replication in the absence of tail amputation. The fates of BrdU-labeled cells in regeneration blastema were assessed 1 (n = 5) and 2 (n = 5) weeks after tail amputation. Serial transverse and longitudinal cryosections (20–30 µm thick) were mounted on glass slides, and air-dried at room temperature. Slides were immersed in 0.1 M PBS for 5 min followed by incubation in HCl solution (1.5 mL HCl/23.5 mL ddH_2_O) for 30 min. at 37°C. Slides were rinsed in PBS, incubated in.1 M sodium borate (pH = 7–8) for 10 min at room temperature, and then immersed in 1% blocking solution (Invitrogen**)** for 30 min prior to incubation in mouse anti-BrdU antibody (BD biosciences, San Jose, CA; 1∶20) for 15–20 h at room temperature. Slides were rinsed in PBS and incubated in fluorescein-conjugated secondary antibody (Alexa Fluor 488 or 546; dilution, 1∶200; Molecular Probes, Eugene, OR) for 1 hr. Sections were again rinsed in PBS, counterstained in propidium iodide or SYTOX, rinsed in deionized water, and cover-slipped with Vectashield Hard Set mounting media (Vector) or Gelmount (Biomeda).

### Intracellular Dye Injections

To test the incidence of cell dedifferentiation during regeneration in *S. macrurus*, fluorescent lineage tracer lysinated tetramethylrhodamine (Fluororuby; 10,000 MW; Molecular Probes, Inc.) was pressure-injected with glass micropipettes and a picospritzer into individual electrocytes or muscle fibers (n = 10–15/fish) of a total of 22 fish under a Zeiss Axiovert 100 M fluorescent microscope. A flap of skin was reflected to allow visualization and access to individual cell types. Fluororuby exhibits low toxicity and is retained in cells for periods of up to 3–4 weeks with little incidence of diffusion [34]; [35]. Tails were transected immediately after muscle fibers and electrocytes were microinjected with the dye and cell filling with the dye verified under a fluorescent scope. At 1-, 2-, 3-, 4-, 5- and 6-days post-amputation, proximal stumps were processed for cryosectioning and immunolabeling with Pax7 and MF20 antibodies using methods described above.

### Image Analysis

Images of immunolabeled tissue sections were visualized and captured on a BioRad 1024 confocal microscope (Bio-Rad Laboratories, Richmond, CA), controlled by OpenLab imaging software (Improvision, Lexington, MA). To quantify Pax7-positive cells in control versus regeneration, images from separate samples of proximal tissue at day 7 (n = 4), control (n = 3) and day 7 blastema (n = 3) were sampled using ImageJ. In each image, three different 100 µm^2^ areas with highest Pax7-positive density were converted to 8-bit and the threshold was modified to reflect positively labeled cells. For proximal and control samples, cells were manually counted using the cell counter plugin. Day 7 blastema was counted using the analyze particle function and the results were displayed to validate correct counts. Manual counting produced the same numbers as produced by the analyze particle plugin. Counts for each image were averaged and the average value from the set of images for each experimental condition were graphed with error bars representing the standard deviation. To quantify colocalization of Pax7 and BrdU, day 7, day 14, and control (n = 3) images were used. For each image, red and green channels were converted to 8-bit and the JACoP plugin [36] was used to calculate Pearson’s coefficient.

## Results

### S. macrurus Pax7 Protein is Highly Conserved

**Figure 2:**
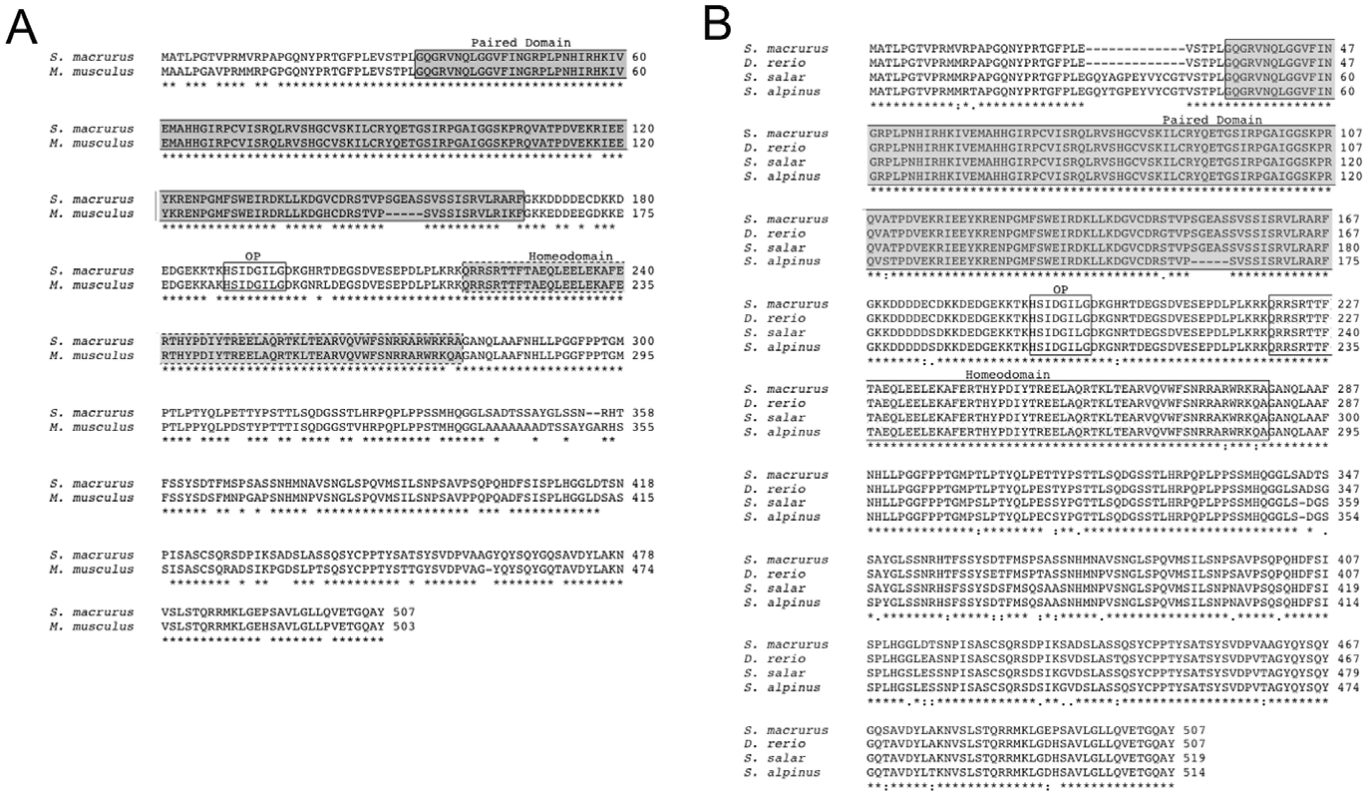
S. macrurus Pax7 protein is highly conserved. Sequence comparison of the deduced protein encoded by S. macrurus Pax7 (GenBank accession number ACC86106) with comparable sequences from mouse (**A**) and other piscine species (**B**). Protein sequences from mouse (M. musculus, GenBank accession number NP_035169.1), zebrafish (D. rerio; accession number NP_571400), Atlantic salmon (S. salar; accession number CAF02090.1), and arctic char (S. alpinus; accession number CAG25716.1) were used. The boxed areas are labeled to indicate the highly conserved regions of members of the Pax family: the paired domain, octapeptide, and homeodomain. Asterisks represent amino acid similarities across sequences.

PCR-coupled RACE generated two Pax7 cDNA sequences from *S. macrurus* skeletal muscle and electric organ tissues: a 2,187 bp cDNA (GenBank accession number EU624121.1) and a 1,809 bp cDNA (GenBank accession number EU624122.1). The longest cDNA contains a 507 amino acid open reading frame that is flanked by a 216 bp 5′ UTR and a 447 bp 3′ UTR. Multiple sequence alignments of the predicted *S. macrurus* Pax7 protein with mouse (accession number NP_035169.1; Figure 2A), and other teleosts including zebrafish (*D. rerio*; accession number NP_571400), Atlantic salmon (*S. salar*; accession number CAF02090.1), and arctic char (*S. alpinus*; accession number CAG25716.1) (Figure 2B) revealed a high degree of conservation among these Pax7 amino acid sequences. The similarity between Pax7 of *S. macrurus*, mouse, and other piscine species ranged from 87% (mouse) to 96% identity (*D. rerio*) (Figure 2). Vertebrate Pax7 contains distinct protein regions that are required for transcriptional activation of its target genes, i.e., the paired box domain, the octapeptide, and the homeodomain (Figure 2A; 37; 38). Within the paired box domain, *S. macrurus* Pax7 showed a 93% homology compared to the mouse protein (Figure 2A), and 98–100% homology to that of other teleosts (Figure 2B). The octapeptide was 100% similar to both mouse and teleost regions (Figure 2), and the homeodomain region of *S. macrurus* Pax7 differed only at one of sixty amino acids (98%) with the other vertebrate Pax7 sequences. In sum, *S. macrurus* Pax7 protein exhibits a high degree of sequence conservation in the specific amino acids that are important for its transcriptional activation. The shorter Pax7 variant contains a 300 amino acid open reading frame that is flanked by a 216 bp 5′ UTR and a 689 bp 3′ UTR (data not shown). The sequences of the three functional protein domains, the paired box domain (100%), the octapeptide (100%), and the homeodomain (78%) were highly conserved between the two Pax7 variants in *S. macrurus*. Compared to the longer variant, the shorter Pax7 variant was truncated downstream of the homeodomain resulting in a shorter putative protein by 207 amino acids.

### Pax7 Expression in Adult Tissues

**Figure 3:**
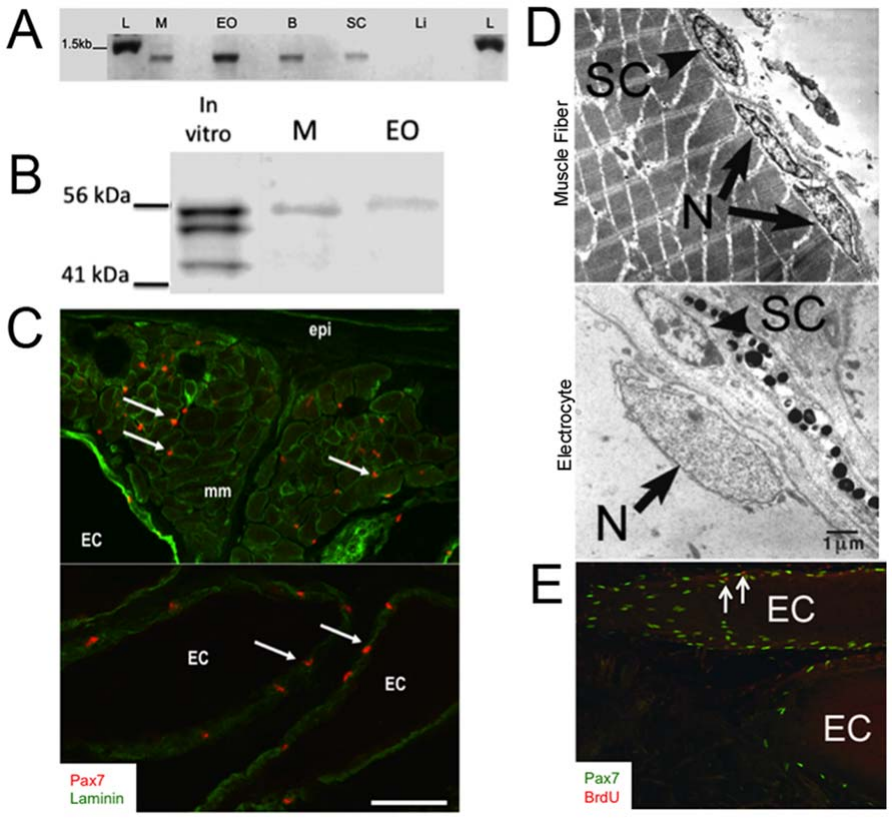
Pax7 labels myogenic satellite cells in adult skeletal muscle and electric organ of S. macrurus. (**A**) Pax7 RT-PCR shows expression in skeletal muscle (SM), electric organ (EO), brain (B), spinal cord (SC), but not liver (L). Negative controls for each sample omitted reverse transcriptase (nrt). (**B**) Pax7 antibody detects in vitro translation products from partial Pax7 cDNA sequence (1,435 bp) and expected band (∼55 kDa) in homogenates (20 µg/lane) from control muscle (M) and electric organ (EO). (**C**) Pax7-positive cells (red) co-label with laminin (green) in tissue cryosections (20-µm thick). (**D**) Electron micrograph cross-sections of adult intact tail show satellite cells (SC) unlike other nuclei (N) are found between the plasma membrane (arrow) and basal lamina in both electrocytes and muscle fibers. (**E**) Few Pax7 cells (green) colabel with BrdU (red) in control longitudinal sections (20-µm thick) (2 Arrow points to co-localization of Pax7 and BrdU. Abbreviations: EC, electrocyte; mm, muscle fiber; epi, epidermis. Scale bars: 50 µm.

We used RT-PCR and immunolabeling to compare the tissue-specific patterns of Pax7 mRNA and protein expression, respectively, in adult *S. macrurus* tissues. Pax7 transcript was detected in mature skeletal muscle, electric organ, brain, and spinal cord tissues, but not in liver (Figure 3A). This tissue expression pattern in *S. macrurus* is consistent with that reported in other vertebrates [39], [30]. *In vitro* translation of a partial 1,435-bp Pax7 cDNA transcript generated protein products that immunoreacted with the mouse monoclonal Pax7 antibody obtained from the Developmental Hybridoma Bank (Figure 3B). A band corresponding to the predicted *S. macrurus* Pax7 protein size of ∼55 kDa was strongly labelled and two smaller bands of sizes between 44–55 kDa were also detected (Figure 3B). All bands detected correspond to sizes that are within the range in Pax7 protein sizes found in mouse (∼55 kDa; accession number P47239), and other teleosts including zebrafish (∼56 kDa; accession number NP_571400.1), arctic char (∼55 kDa; accession number Q5ZNB2), and Atlantic salmon (∼56 kDa; accession number Q683Y9). Western blots using *S. macrurus* control skeletal muscle and electric organ tissues revealed a single band of approximately 55 kDa. These data provide support for the specificity of the mammalian Pax7 antibody against *S*. *macrurus* Pax7 protein. It was not determined whether the multiple *in vitro* translation products were due to internal translation initiation of the cDNA. However, proteins of approximately 54, 53, 49, and 43 kD could be explained by leaky initiation of translation at the first four internal in-frame ATG residues of the *S. macrurus* Pax7 mRNA. On tissue sections, Pax7 co-localized with laminin immunolabeling in both muscle fibers and electrocytes (Figure 3C). Ultrastructural analysis using electron micrographs of longitudinal and cross sections from muscle and electric organ of adult tails corroborated the location of mononuclear cells beneath the basement membrane of muscle cells and electrocytes (Figure 3D). In control tails, the incidence of Pax7-positive cells that incorporated BrdU was relatively low (Figure 3E). By virtue of their Pax7 expression, morphology, and location, these cells associated with muscle fibers and electrocytes can be considered to be homologs of satellite cells observed in mammals [40], [41], salamanders [9], frogs [42], and other teleosts [43] and birds [44].

#### Spatial distribution of Pax7-positive cells in regeneration blastemas

Characterization of the tail regeneration process after tail amputation in *S. macrurus* has been described in previous studies [21], [28], [31]. Briefly, when the tail is amputated at the end of the ventral fin, epidermal cells at the wound margin rapidly proliferated and covered the wound within 24 hr. After one week, blastemas reached an average length of 4 mm (range: 2.2–4.8 mm; SD: ±1.1) and were composed primarily of undifferentiated mesenchymal cells. During the second week, blastemas grew to an average length of 7 mm and contained spinal neurons, muscle fibers and electrocytes at different stages of differentiation. Muscle fibers formed adjacent to the epithelium with some of the larger muscle cells farthest from the epithelium fusing and converting into electrocytes – data that supports previous observations in *S. macrurus* tail regeneration [28], [29], [31].

**Figure 4:**
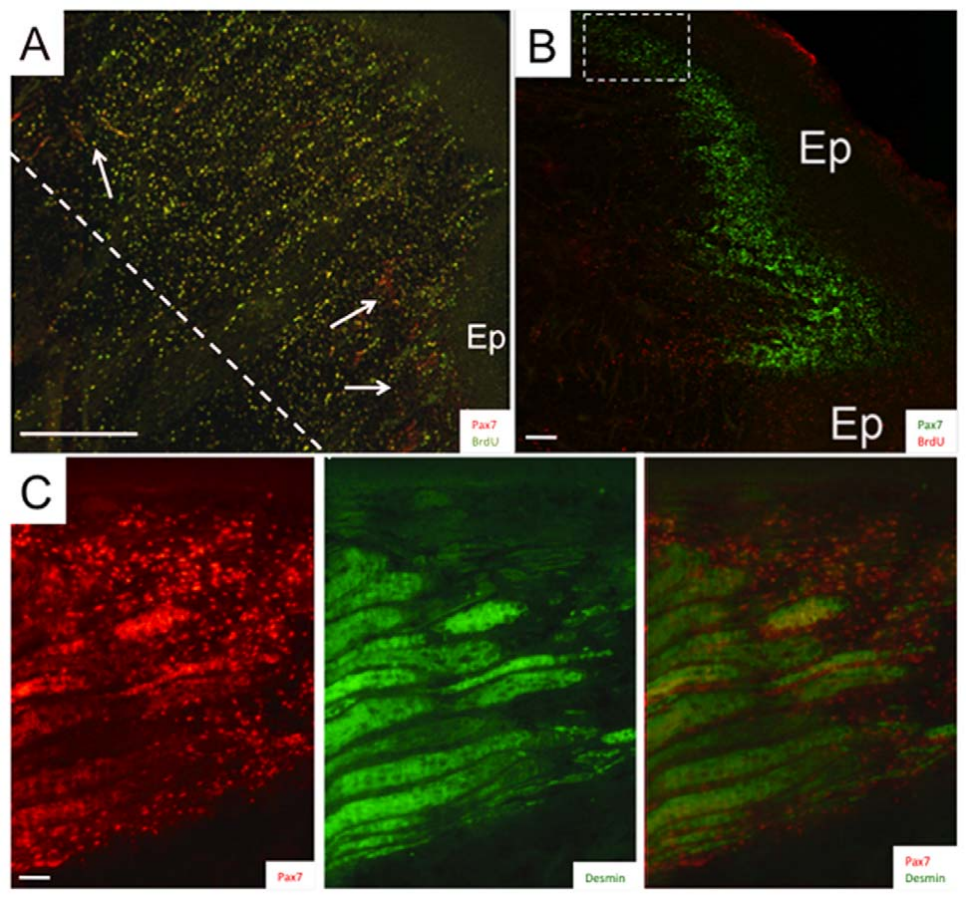
Spatial distribution of Pax7-positive cells in regeneration blastema. Confocal images of longitudinal cryosections (20-µm thick) from 7-day (**A**) and 14-day (**B**) blastemas immunolabeled with anti-Pax7 (**A**, **B**, **C**), BrdU (**B**) and desmin (**C**) antibodies. Dashed line in **A** denotes the site of amputation with the newly regenerated blastema to the right of the amputation site. Arrows point to electrocytes damaged by amputation. Arrowheads point to Pax7-positive cells adjacent to the epithelium (Ep). Pax7-positive cells are red in **A** and **C**, and green in **B**. Images in **C** represent the region enclosed in the dotted box in **B** from a serial cryosection co-labelled with anti-desmin and Pax7 antibodies. Scale bars: **A**, 400 µm; **B**, 150 µm; **C**, 50 µm.

**Figure 5:**
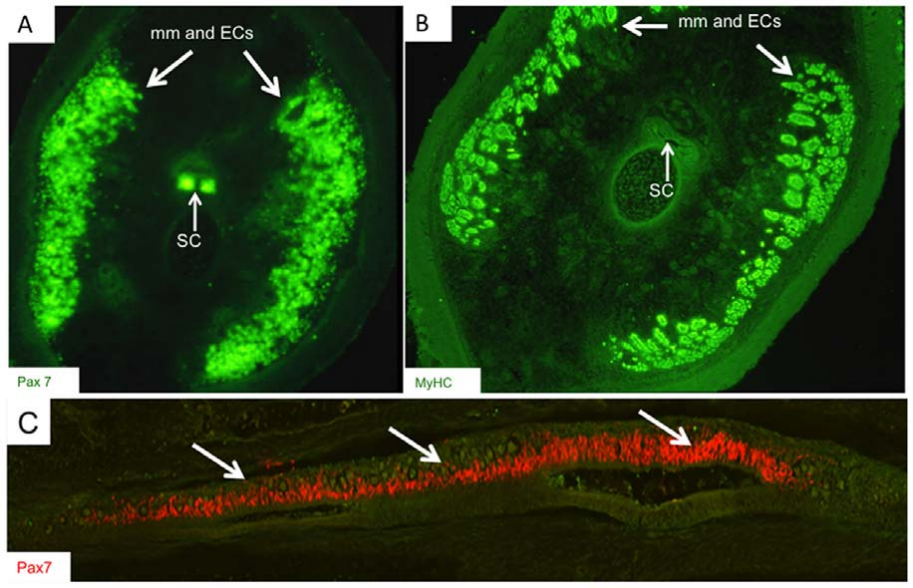
Pax7-positive cells are localized in blastema regions that give rise to muscle, electric organ and dorsal spinal cord. Serial cross-sections taken from distal half of 14-day blastemas (**A** and **B**) immunolabeled with antibodies against Pax7 (**A**, green) and MF20 (**B**, green). MF20 labels all mammalian myosin heavy chain (MyHC) isoforms present in all muscle fibers and developing electrocytes that arise from the fusion of muscle fibers. Immunolabeling by Pax7 and MF20 was detected in peripheral regions of the blastema underneath the epithelium. Pax7 immunolabel was also detected in cells within the dorsal spinal cord of regenerating blastema (**A**, arrowhead). **C**, Pax7 immunolabeling was detected along the entire regenerating dorsal spinal cord in 14-day blastemas as shown in this longitudinal section. Abbreviations: ECs, electrocytes; mm, muscle fibers; SC, spinal cord. Scale bars: **A**, 300 µm; **B**, 300 µm; **C**, 150 µm.

To determine whether Pax7-positive cells are associated with the restoration of myogenic cells in the blastema, we studied the expression and distribution of Pax7 at different stages of regeneration. Within the first week after amputation, most Pax7-positive cells were detected peripherally underneath the epithelium of the blastema (arrowheads, Figure 4A). This distribution was also true for 2-week blastemas, i.e., Pax7-positive cells were abundant and highly concentrated beneath the epithelium (Figure 4B). Blastema regions under the epithelium correspond to those that give rise to myogenic cells as evident by co-labeling with antibodies against muscle markers desmin (Figure 4C) and sarcomeric myosin heavy chain (Figure 5B). The co-localization of Pax7-positive cells within developing muscle fibers and electrocytes (Figures 4C and 5A-B) clearly demonstrate the contribution of Pax7-positive cells to the formation of the same myogenic tissues they are associated with in adult intact tails. Outside of these myogenic zones in the blastema, Pax7 immunolabeling was only detected in cells along the entire antero-posterior axis of the regenerating spinal cord (Figure 5A, C). The occurrence of Pax7-positive cells exclusively in the dorsal zones of the spinal cord in blastema and adult tails of *S. macrurus* is consistent with expression patterns reported in developing and regenerating spinal cords in other vertebrates [37], [39], [45]–[47]. In sum, our data support a mechanism of regeneration that is mediated primarily through a population of Pax7-positive stem cells that give rise to dorsal spinal cord, muscle fibers and electrocytes.

#### Pax7 expression increases in tissues proximal to the amputation site

**Figure 6:**
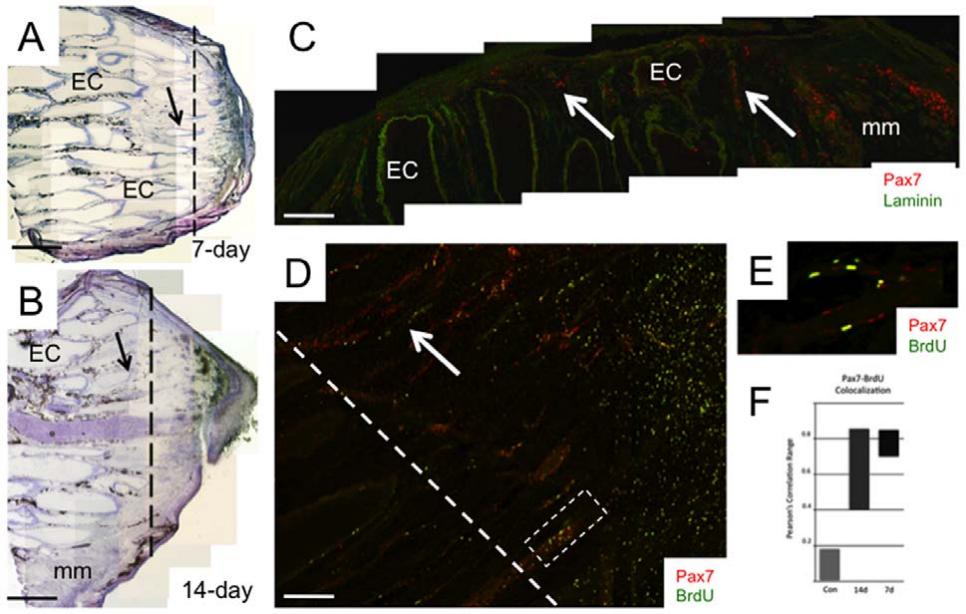
Degeneration of electrocytes injured by amputation. Portions of longitudinal cryosections (14–30 µm thick) from 7-day (**A**) and 10-day (**B**) blastemas stained with hematoxyline and eosin. (**C**) 7-day blastema co-labeled with anti-Pax7 (red) and anti-laminin (green) antibodies. (**D**) 7-day blastema co-labeled with anti-Pax7 (red) and anti-BrdU (green) antibodies. Dashed line in **A**, **B** and **D** show the sites of tail amputation. Arrows in **A**, **B**, **C** and **D** point to electrocytes that were damaged by the amputation. **E**, Enlargement of the region within the dashed area in **D** to more clearly visualize the colocalization of Pax7 (red) and BrdU (green) as yellow. **F**, Correlation of Pax7 and BrdU immunolabeling in cells from control tails (Con), and tails after amputation at 7 (7 d) and 14 (14 d) days. Abbreviations: EC, electrocyte; Ep, epithelium; mm, muscle fiber. Scale bars: **A**, 2 mm; **B**, 5 mm; **C**, 500 µm; **D**, 200 µm. **F**, Range of Pearson’s correlation values for Pax7/BrdU co-localization for images from 3 different tails taken from control fish, and 7-day and 14-day amputated tails.

**Figure 7:**
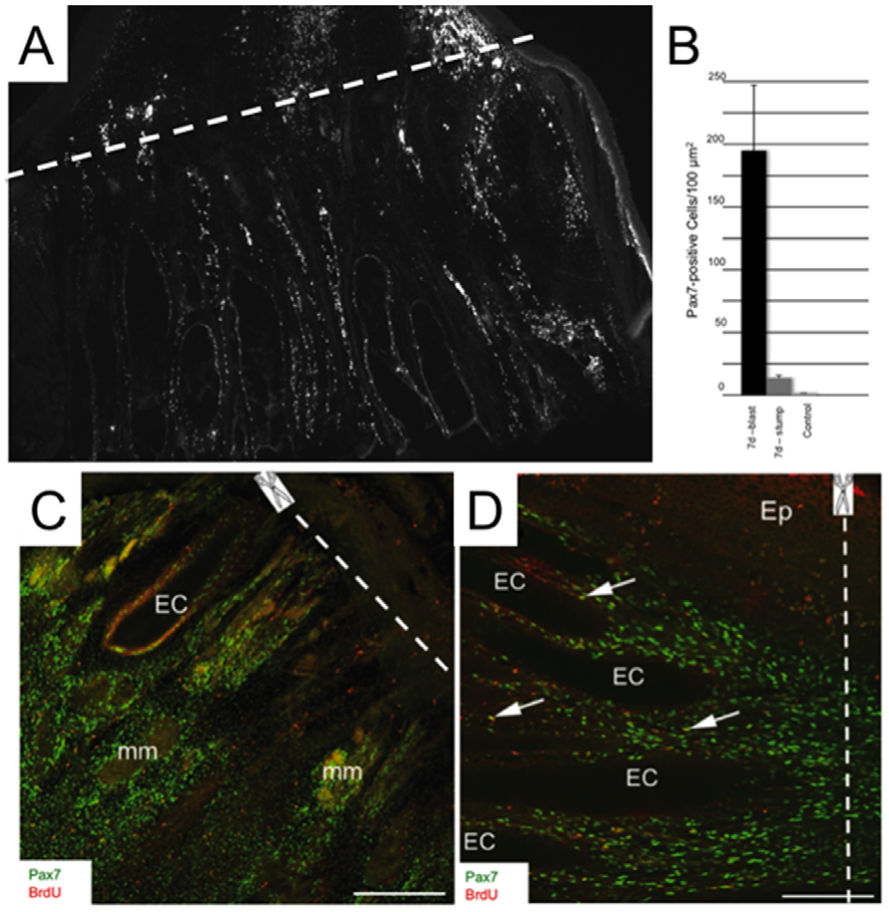
Pax7 expression increases proximal to the amputation site. A , Black and white image of a portion of a longitudinal cryosection (20-µm thick) of a 7-day tail immunolabeled with the antibody against Pax7. Proximal stump is the region below to the dashed line and regeneration blastema is the region above dashed line. **B**, The density of Pax7-positive cells is increased in the proximal stump (>8×) (stump) and regeneration blastema (>100×) (blast) compared to control, based on images from 4 different tails taken from control fish, and 7-day amputated tails. **C** and **D**, Portions of longitudinal sections (20-µm thick) of 14-day amputated tails immunolabeled with antibodies against Pax7 (green) and BrdU (red). Dashed lines denote the site of amputation with the proximal stump to the left. Arrows point to cells that were co-labeled with Pax7 and BrdU. Abbreviations: EC, electrocyte; Ep, epithelium. Scale bars: **A**, 250 µm; **B**, 100 µm.

Changes in Pax7 expression were determined at different time periods after amputation in the tail region proximal to the amputation site, a tail segment that included both injured and intact cells (Figure 6). Electrocytes injured by tail transection were easily distinguished from intact electrocytes based on their staining with hematoxylin and eosin (Figure 6A–B). Similar distinctions among muscle fibers before and after injury were not made due to their much smaller size and number. Injured electrocytes atrophied into long thin strips that extended into the regeneration blastema (Figure 6A–B). At seven days after amputation, these thin strips of damaged electrocytes contained Pax7-positive cells (Figure 6C–D). BrdU incorporation studies showed higher Pearson’s correlation r values for colocalization of Pax7 and BrdU (Figure 6E) in tails from both 7 and 14 days after amputation compared to tails from control uncut fish (Figure 6F), providing further support that they are the product of a recent cell division. The longitudinal cryosections shown in Figure 7 are representative of the density of Pax7-positive cells proximal to the amputation site observed in all fish studied. We observed an increase in the density of Pax7-positive cells associated with electrocytes and muscle fibers in the proximal stump seven days after tail amputation (Figure 7A) that was >8× higher than that in control uncut tails (Figure 7B). The increased density of Pax7-positive cells in blastema was >100× higher than found in control tissues (Figure 7B). By day 14 post-amputation, most Pax7-positive cells occupied the extracellular space (Figure 7C–D) a distribution not observed in control intact tails (Figure 3C).

#### Injury-dependent responses of muscle and Pax7 expression

**Figure 8:**
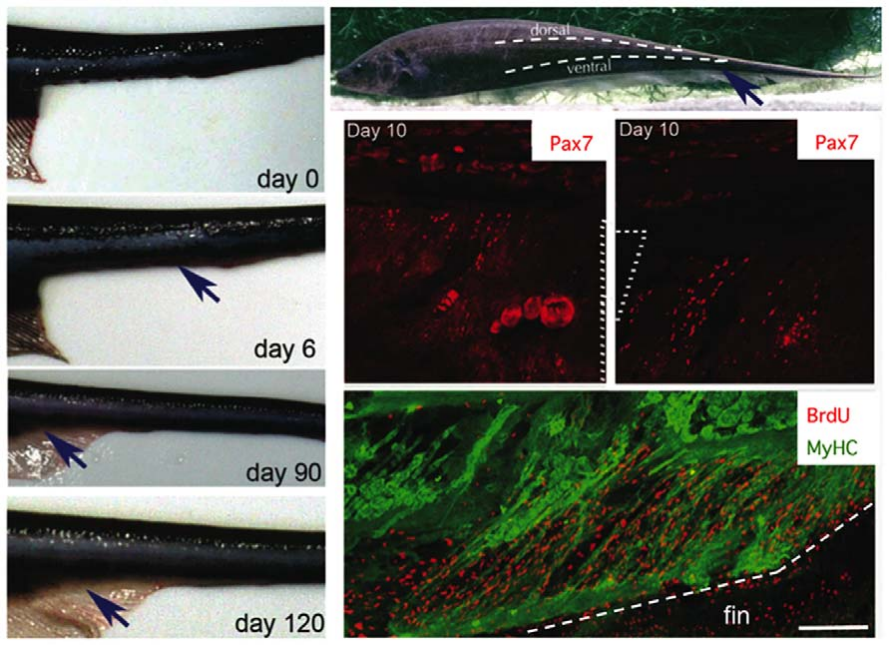
Delayed regeneration of ventral muscle associated with fewer Pax7-positive cells. An adult S. macrurus and delineation of its ventral and dorsal muscle groups. Four images show the regeneration of the most distal portion (2 to 3 cm) of the ventral muscle over a 120-day period after its ampuation. Blue arrow points to the distal ventral muscle region removed including portions of the ventral fin. Serial longitudinal cryosections of a tail 10 days after ventral muscle excision were immunolabeled with anti-Pax7 (red) antibody to show the incidence of Pax7 immunolabeling at two different depths of the tail region adjacent to the muscle excision area. Dotted lines on the top two Pax7 images represent site of ventral muscle excision. The bottom panel shows longitidunal section from the same animal immunolabeled with anti-BrdU (red) and MyHC (MF20; green) antibodies. A dotted line has been added to the bottom image (BrdU/MyHC) to clearly discern the boundary between skeletal muscle and fin adjacent to the site of excision. MyHC is not expressed in the fin. Scale bar for Pax7/BrdU immunolabeled images: 200 µm.

To further investigate the regenerative potential of skeletal muscle Pax7-positive cells in *S. macrurus*, a section of ventral muscle was amputated. In this procedure, electric organ, central blood vessel and spinal cord were left intact. Pax7 immunolabeling and BrdU incorporation studies were done in the same way as with studies after tail amputation described above. The response to removal of ventral muscle was similar to the process observed after tail amputation, i.e., immediate formation of a wound followed by the formation of a blastema and its growth to give rise to new ventral muscle (Figure 8). Full restoration of all ventral muscle tissue removed was generally completed 20 to 22 weeks after its removal, that is, 15 to 18 weeks longer than when a similar length of muscle was removed after complete tail amputation. Pax7-positive cells were detected within muscle fibers adjacent to the amputation site. However, despite the lack of quantitative assessment, the apparent number of Pax7-positive cells observed in response to tail amputation appeared higher than that observed in response to ventral muscle amputation. This difference is visibly evident in Figures 7 and 8 wherein the incidence of Pax7-positive cells within a 200 µm segment of tissue in tails after amputation (Figure 7) is higher compared to that within a 200 µm segment of tissue after ventral muscle amputation (Figure 8). BrdU incorporation was observed but mainly within the fin and not in muscle fibers adjacent to the injury sites (Figure 8). These data could be explained by an injury response that involves a differential potential in the activation of Pax7-positive cells under different injury scenarios in *S. macrurus* – a hypothesis that will be tested in future studies.

### No Evidence for Myogenic Cell Fragmentation after Tail Amputation

**Figure 9:**
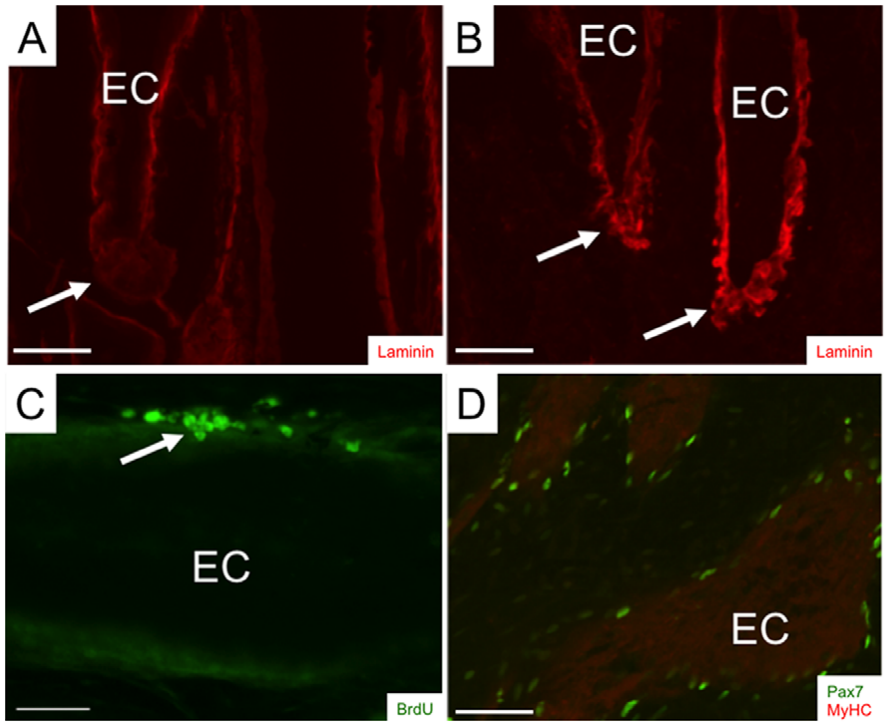
No evidence of cell dedifferentiation. Portions of longitudinal cryosections (14-µm thick) from intact tails (**A** and **D**) and tails 4 (**B**) and 14 days (**C**) after amputation. Tissue sections were immunoreacted with anti-laminin (**A** and **B**) to study changes in basal lamina morphology that may indicate cellularization (arrows), anti-BrdU (**C**) to investigate myonuclei proliferation after tail cut (arrow), and anti-myosin heavy chain (**D**, red) to examine changes in muscle protein expression in intact mature electrocytes proximal to the tail transection site. Sections in **D** were co-immunolabeled with anti-Pax7 (green). Abbreviations: EC, electrocyte. Scale bars: 50 µm.

The occurrence of cell dedifferentiation has been a major focus in urodele limb regeneration studies. Morphological and cell tracing experiments, for instance, have demonstrated that mature skeletal muscle fibers can fragment (or cellularize) into smaller nuclei-containing units, proliferate, and contribute as a source of new cells that make up the blastema [48]–[51]. In this study, when we stained longitudinal tissue sections with hematoxylin and eosin (Figure 6A–B) or immunolabeled them with anti-laminin (Figure 9A–B), we did not observe changes in cell morphology of muscle fibers and electrocytes proximal to the transection site that resembled cell fragmentation. Similarly, our BrdU incorporation studies did not reveal labeling of nuclei inside muscle fibers or electrocytes (Figure 9C) showing that these cells did not dedifferentiate via reentry into the cell cycle. Instead, BrdU incorporation observed was in cells outside the plasma membrane of muscle fibers and electrocytes (Figure 9C, arrows). There were few intact electrocytes that were immunolabeled with the anti-sarcomeric MHC antibody MF20 (Figure 9D) manifesting an earlier stage in their differentiation from skeletal muscle fibers. However, these MF20-positive electrocytes did not show any other evidence of a dedifferentiation process beyond the re-expression of MyHC.

**Figure 10:**
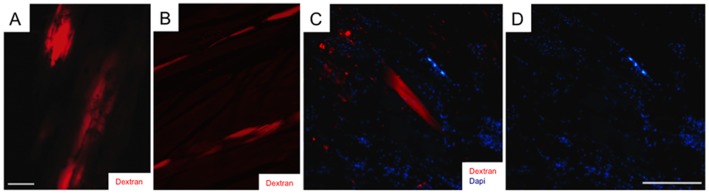
Intracellular tracer dye injections into single muscle fibers and electrocytes at the level of the distal-most ventral fin reveal no cell fragmentation. Fluororuby (red) was injected into single electrocytes (**A**) and muscle fibers (**B**) and visualized 7 days after injection by retracting the overlying skin of the fish. Tail sections at the level of the distal-most region of the ventral fin were processed for immunolabeling and viewed under a fluorescent microscope. (**C**), region of a cryosection containing muscle fibers injected with Fluororuby dextran (red) and counterstained with DAPI (blue). (**D**), same image as C showing only DAPI labeling. Scale bars: **A** and **B**, 20 µm; **C** and **D**, 10 µm.

To more rigorously test the incidence of cell fragmentation of myogenic tissues during regeneration in *S. macrurus*, fluorescent dextran was injected into single electrocytes and muscle fibers prior to tail amputation. Longitudinal cryosections of the distal-most region of the ventral fin (50 mm from amputation site) were processed for detection of dextran during the first two weeks after tail amputation. Our *in vivo* dye-injection studies showed no evidence of cell fragmentation in any of the tails analyzed up to 14 days post-amputation. Electrocytes labeled with single-cell dextran injections showed an intact morphology with no fragmentation into smaller cellular components (Figure 10A). Similar results were obtained with dextran-injected muscle fibers (Figure 10B–C). Small fluorescent remnants of dextran labeling were at times detected around some electrocytes and muscle fibers (Figure 10C), but this fluorescence was not co-labeled with nuclear stain DAPI (Figure 10C–D) indicating these were acellular debris generated by cutting labeled cells. Hence, at least with regard to data from laminin labeling and intracellular dye injection experiments, multinucleated muscle fibers and electrocytes proximal to the injury site did not undergo fragmentation. Hence, these findings are contrary to the association between blastema formation and dedifferentiation of cells near the injury site that has been reported in urodeles [6], [48]–[51].

## Discussion

Our cloning analysis revealed that specific amino acids important for transcriptional activation by Pax7 in mammals are also highly conserved in the *S. macrurus* ortholog (Figure 2). A common feature of the Pax7 gene is the generation of different transcripts through alternative splicing [37]–[39]; [52]–[53], a feature confirmed for the Pax7 gene in *S. macrurus*. We identified 2 alternate Pax7 isoforms (GenBank accession numbers EU624121 and EU624122), which varied in size from 300 to 507 amino acid residues, a range similar to that found among Pax7 isoforms reported within different vertebrate species. The number of Pax7 variants confirmed in *S. macrurus* is fewer than the number of alternate Pax7 transcripts identified in other vertebrates, which range from at least four variants in mice [38]–[39] and zebrafish [37] to at least 10 variants in other teleosts including Atlantic salmon [52] and the arctic charr [53]. Hence, it remains to be determined whether the Pax7 gene of *S. macrurus* encodes additional isoforms, an issue that was beyond the scope of the present study. In addition, establishing whether alternate transcripts are expressed in different cell populations in *S. macrurus* as it is known in mice and other teleosts would suggest different tissue-specific roles of Pax7 function that are highly conserved in vertebrates.

Our RT-PCR and immunolabeling studies also suggest that expression of Pax7 mRNA and protein, respectively, is highly conserved in *S. macrurus*. Pax7 transcripts in *S. macrurus* were mainly found in mature skeletal muscle, electric organ, brain and spinal cord where Pax7 expression was restricted to a subpopulation of neural cells in the dorsal spinal cord. These Pax7 expression patterns in skeletal muscle, brain and spinal cord are similar to those in adult mouse [30], [38], urodele amphibians [46] and other teleosts [52]–[53]. We also show that the commonly used mammalian antibody against Pax7 from the Developmental Hybridoma Bank labeled protein products from *in vitro* translated Pax7 cDNA and whole tissue homogenates from muscle and electric organ of *S. macrurus* that correspond to the expected sizes of known Pax7 proteins in mammals and other teleosts (Figure 3B). More importantly, cells associated with muscle fibers and electrocytes with the morphology and location similar to those that define myogenic satellite cells were immunolabeled with the Pax7 antibody (Figure 3C). By combining nuclear Pax7 expression with morphological observations of satellite cells by electron microscopy we conclude that Pax7 is a reliable satellite cell marker in *S. macrurus* muscle and electric organ. Other molecules like c-Met and M-cadherin have been used to identify satellite cells by other investigators [54]–[55]. However, unlike the Pax7 antibody, antibodies against these other markers did not cross-react successfully with *S. macrurus* tissues. Pax7 is a commonly used marker for identifying quiescent satellite cells [30], [56]. In control intact fish, Pax7-positive cells associated with electrocytes and muscle fibers rarely incorporated BrdU (Figure 3E and Figure 6E) suggesting a predominant state of quiescence. Following tail amputation, only some Pax7-positive cells proximal to the amputation site were BrdU positive, and the low incidence of Pax7-BrdU-positive co-labeling was similar regardless of whether satellite cells were associated with, or free of, electrocytes and muscle fibers (Figure 7). These data may reflect the BrdU injection paradigm used for this timepoint wherein, in all likelihood, was not incorporated into all cells that re-entered cell division in response to tail amputation.

Since the first description in frog muscle by Mauro in 1961 [57], satellite cells have been identified in many vertebrate species and have been considered distinct progenitor cells with the unique ability of associating with, generating, repairing, and maintaining skeletal muscle. This muscle-specific fate has recently been challenged by studies showing that satellite cells can adopt different cell phenotypes [58]–[61]. In each of these cases, satellite cells have been taken away from their natural niche and cultured under conditions to promote the conversion of satellite cells into cells with adipocyte and osteocyte [58]–[60], and even some neuronal characteristics [61]. Yet, we are not aware of any studies wherein satellite cells show this cell fate potential *in vivo* and/or demonstrate the occurrence of satellite cells in mature fat, bone or neuronal tissues. In contrast, the association of satellite cells with mature electrocytes is a natural occurrence that represents an exception to the tissue-specificity of these myogenic stem cells to striated muscle fibers. A major quest of future studies is to determine whether the Pax7-positive cells in muscle and electric organ of *S. macrurus* form a single multipotent class of cells or whether they represent discrete satellite cells for muscle and electrocyte lineages. One way to explore this is to replicate differentiation of muscle fibers and electrocytes in culture. Our ability to isolate satellite cells from muscle fibers or electrocytes will allow us to directly test the potential of these myogenic cells under suitable differentiation conditions [62]–[63]. It will be interesting to find out if satellite cell populations isolated from either tissue reflect their cell type of origin as has been observed with satellite cell populations that give rise to either slow or fast muscle fibers in birds and rodents [64]–[66] – or not, as it has been reported in humans [67]. Our present characterization of satellite cells in muscle and electric organ of *S. macrurus* represent a modest beginning of a large-scale project that ultimately includes understanding how the skeletal muscle phenotype is transformed to acquire new functions and how this may occur independently in multiple lineages in teleosts.

We studied the response of Pax7-positive cells following amputation, blastema formation, and regeneration of muscle fibers and electrocytes. Upon tail amputation, Pax7 positive cells associated with intact electrocytes and muscle fibers proliferated profusely (Figure 7). Pax7-positive cells associated with electrocytes and muscle fibers that were damaged by tail amputation were also observed to extend in narrow strip-like formations into the blastema (Figures 3A, 5C–D). Within the blastema, the incorporation of Pax7-positive cells into developing dorsal spinal cord, muscle fibers and electrocytes was unmistakable (Figures 4 and 5). This cellular distribution corresponds to tissues which Pax7-positive cells manifest in adult intact tails of *S. macrurus* (Figure 3). Hence, with regards to the incorporation of Pax7-positive cells into myogenic tissues, our results demonstrate that muscle and electrocyte regeneration in adult *S. macrurus* is similar to the satellite-dependent muscle repair process common in vertebrates including mammals, frogs, birds, fish and some salamanders [9,55–56,å68–70]. That skeletal muscle regeneration in *S. macrurus* shows considerable dependence on activation of myogenic Pax7-positive stem cells is further supported by results obtained from our ventral muscle amputation experiments that showed a slower regeneration process associated with considerably fewer changes in Pax7-positive cell number (Figure 8) compared to that observed after tail amputation (Figures 5 and 7).

It has been well documented that formation of a blastema can result from the dedifferentiation of mature tissues proximal to the amputation site [6]–[7], [71]. Muscle dedifferentiation can involve the reentry of myonuclei into the cell cycle, cellularization or fragmentation of muscle fibers, and/or the loss of muscle-specific proteins, such as, sarcomeric proteins. Our morphological analysis of muscle fibers and electrocytes did not detect evidence of fragmentation of these myogenic cells that was indisputable when compared to control muscle and electrocytes (Figure 9). Our experiments also do not support the occurrence of myonuclei reentering the cell cycle since BrdU incorporation was only detected in satellite cells (Figure 9C). We did observe a few intact electrocytes near the amputation site that re-expressed sarcomeric myosin heavy chain (Figure 9D) indicating a partial reversal in their myogenic phenotype. However, our single-cell tracer dye injection experiments provide the strongest evidence against *in vivo* fragmentation or cellularization of either muscle fibers or electrocytes in response to tail amputation (Figure 10). Data from these intracellular dye injection experiments together with our Pax7 expression studies suggest that skeletal muscle and muscle-derived electrocytes are regenerated in *S. macrurus* by a mechanism similar to the myogenic stem cell-dependent muscle repair process seen in mammals. Hence, muscle dedifferentiation is likely one regeneration strategy, but not the requirement in the myogenic lineage of the adult teleost *S. macrurus*. In sum, this first characterization of the cellular mechanisms involved in the regeneration of myogenic tissues of the adult tail in *S. macrurus*: 1) demonstrates the important implications that studying regeneration in different animals with robust regeneration potential have on our understanding of the evolution of regeneration processes, 2) support the emergent concept in vertebrate regeneration that different tissues provide a distinct progenitor cell population to the regeneration blastema, and these progenitor cells subsequently restore the original tissue [10], and 3) represent an exciting starting point in the comparative investigation of regeneration mechanisms underlying the restoration of myogenically derived, highly specialized non-contractile tissues in other teleosts.
